# Improved Survival With Surgical Treatment of Primary Lung Lesions in Non-Small Cell Lung Cancer With Brain Metastases: A Propensity‐Matched Analysis of Surveillance, Epidemiology, and End Results Database

**DOI:** 10.3389/fonc.2022.888999

**Published:** 2022-07-22

**Authors:** Qing Wang, Jing Li, Xiaohua Liang, Qiong Zhan

**Affiliations:** Department of Oncology, Huashan Hospital, Fudan University, Shanghai, China

**Keywords:** non-small cell lung cancer, brain metastases, propensity score matching, surgical treatment, surveillance, epidemiology, and end results

## Abstract

**Objectives:**

Non-small cell lung cancer (NSCLC) with Brain metastases (BM) is an advanced disease with poor prognosis and low survival rate. Our study evaluated the survival benefit of primary lung resection with mediastinal lymph node dissection in NSCLC patients with BM using Surveillance, Epidemiology, and End-result (SEER) databases.

**Methods:**

All cases analyzed were from Surveillance, Epidemiology, and End Results database. The data of the patients with BM of NSCLC from 2010 to 2016 was retrospectively analyzed. Patients (N=203) patients who underwent radical surgical treatment for primary lung lesions and patients (N=15500) who did not undergo surgery were compared. We successfully analyzed patients using propensity score matching (PSM). Kaplan‐Meier and Cox‐ regression analyses were applied to assess prognosis.

**Results:**

The median survival in the surgery group was longer than in the control group (27 months vs 5 months; P < 0.001) in the overall sample, 21 months longer compared to the control group (27 months vs 6 months; P<0.001) in a PSM cohort. Cox regression analysis showed that underwent surgery patients in the propensity-matched sample had a significantly lower risk of mortality (HR:0.243, 95%CI: 0.162-0.365, P < 0.001) compared with untreated patients. Multivariate analysis identified the following as independent risk factors for NSCLC with BM: no primary resection surgery, age >65 years, worse differentiation, squamous cell carcinoma, lymphatic metastasis, no systemic therapy. Subgroup analysis revealed that radical resection of the primary lung provided a survival benefit regardless of marital status, tumor size, tumor grade, tumor T stage, and mediastinal lymph node metastasis after PSM.

**Conclusion:**

Radical resection of primary lung can improve the survival of NSCLC patients with BM. Male, age>65years, poorly differentiated tumor, tumor size>5cm, and mediastinal lymph node metastasis were factors for poor survival.

## Introduction

Lung cancer is one of the most common malignancies, accounting for about 85% of non-small cell lung cancer (NSCLC), and the 5-year survival rate is only 16% (1–3). NSCLC with BM are widespread, with an incidence of about 30%-50% (4). NSCLC with BM generally has a poor prognosis, and the median survival time of untreated patients is less than one month (5, 6).Despite some therapeutic advances, such as intracranial surgical resection, whole brain radiotherapy (WBRT), stereoscopic radiotherapy (SRS), and chemotherapy, NSCLC patients with BM still have a poor prognosis. In the past, NSCLC patients who had single BM with resectable lung lesions were considered to be clinically at stage IV. Such patients were considered to have no survival benefit after resection of the lung primary tumor, so no active surgical treatment was required for the lung lesions, so chemotherapy or radiotherapy were generally only given.In recent years, it has been found that selective pulmonary resection can improve the prognosis of NSCLC patients with isolated single BM, whose mOS ranges from 20.5 months to 64.9 months (7–10). In addition, whether the primary lung tumor is completely removed is related to postoperative recurrence and prognosis (10, 11). Although NSCLC with BM is at an advanced stage, the principle of radical treatment should still be followed during lung surgery to maximize the removal of tumor tissue and routine dissection of regional lymph nodes, so as to achieve the best therapeutic effect.

With the improvement of diagnosis and treatment, there is an urgent need for more effective treatment to improve the prognosis of patients. The clinical significance of surgical selective resection of pulmonary primary lesions and brain metastases has attracted attention, but the feasibility and effectiveness of surgical treatment for such patients are still controversial. However, few studies have been reported on the resection of lung primary lesion and conventional mediastinal lymph node dissection, and large sample data are lacking. Therefore, the purpose of this study was to evaluate the survival benefits of pulmonary primary resection with mediastinal lymph node dissection in NSCLC patients with BM based on Surveillance, Epidemiology, and end Results databases.

## Materials and Methods

### Ethics Statement

The data about cancer in the SEER database is continually reported in every state of the United States and retrieved with no need for informed patient consent. The present study complied with the Declaration of Helsinki (as revised in 2013).

### Data Source

Data in this population-based study were abstracted from the SEER. SEER*Stat Software version 8.3.4 (https://seer.cancer.gov/seerstat/; Information Management Service, Inc., Calverton, MD, USA) was used to generate the case listing.

### Study Population

Our data came from the National Cancer Institute’s (NCI) SEER database, andbecause SEER did not record BM information until 2010.Patients diagnosed before that year were excluded. Therefore, a retrospective cohort study was conducted on patients diagnosed from 2010 to 2016. SEER is an open access U.S. cancer database from 18 population-based cancer registries. SEER currently collects and publishes cancer incidence and survival data covering approximately 28% of the U.S. population, which is representative of the population.

### Inclusion and Exclusion Criteria

We screened patients from the SEER database with pathologically diagnosed NSCLC, including adenocarcinoma, squamous cell carcinoma, and adenosquamous cell carcinoma, according to the International Classification of Diseases of Cancer Version 3 (ICD‐O‐3) histological codes 8140, 8070, 8560, 8046. Ethical approval and informed consent were waived because the SEER data were freely available and our investigation was retrospective. The exclusion criteria for NSCLC patients in this study were as follows: (I) no BM; (II) patients with multiple primary malignant tumors; (III) patients with NSCLC whose survival was less than one month or whose survival data were not available were excluded. We excluded patients who did not undergo primary pneumonectomy and mediastinal lymphadenectomy, such as local lobectomy, lymphadenectomy, laser ablation, or cryotherapy. Follow-up was from diagnosis of NSCLC to death or the end of the follow-up period.

### Propensity Score Matching (PSM)

The purpose of this study was to compare the benefits of primary lung resection with mediastinal lymph node dissection in patients with NSCLC with BM. This was a retrospective study, so the surgery assignment was not random. Some of the key covariates of patients in the active and control groups were heterogeneous and could have influenced the results. Therefore, we further compared the difference in survival between the surgical and untreated groups by univariate analysis using 1:2 nearest neighbor matching and setting the caliper value to 0.02. The PSM process has been applied to minimize selection bias and roughly balance baseline covariates in an intergroup set of analyses (12).

### Statistical Analysis

The primary endpoint of the study was overall survival (OS). Chi-square test was used to compare the characteristics of surgical patients and control patients. Covariates in this study included multilevel factors (such as age, sex, race, marital status, insurance status, tumor tissue type, tumor size, lymph nodes, degree of differentiation, chemotherapy and radiotherapy). SEER data recorded a small number of tumor lesions larger than 20cm. We thought these might be incredible, so we included them in part to understand the participation statistics. Propensity scores are used to reduce selection bias. Kaplan‐Meier analysis was used to estimate OS before and after PSM. Log-rank tests were performed to compare survival differences in patients, lesions, and treatment-related characteristics. To perform a multivariate analysis in a matched population, we constructed a Cox proportional risk model to identify predictors ofsurvival. P value<0.05 considered that the difference was statistically significant. All statistical analyses were performed using IBM SPSS Statistics 22.0 (IBM, Armonk, NY, USA).

## Results

### Baseline Characteristics

**Figure 1:**
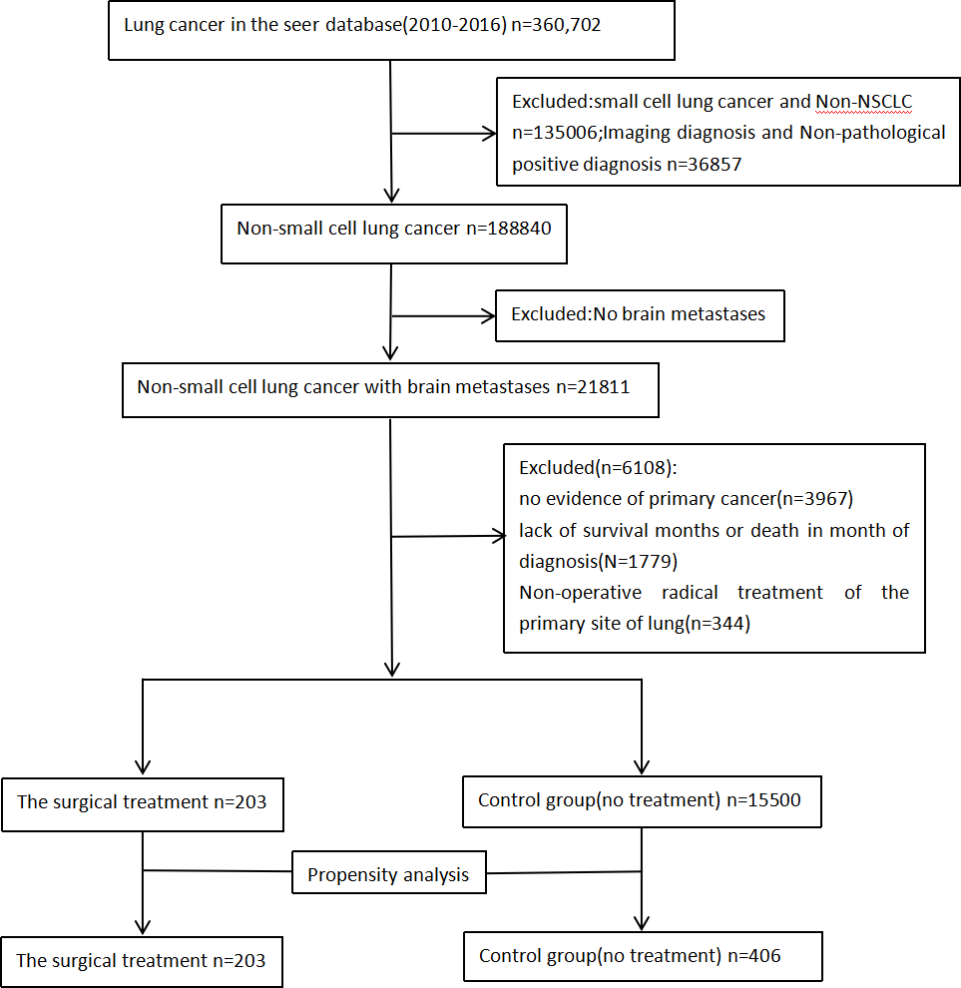
SEER Data extraction and filtering flowchart.

From 2010 to 2016, 188,840 patients with newly diagnosed NSCLC were identified in the SEER data set. A total of 21,811 patients diagnosed with advanced NSCLC with BMs were selected based on inclusion criteria described in the study population. Of these, 15,703 met the inclusion criteria for this study. The surgical and untreated groups included 203 (1.31%) and 15,500 (98.69%) patients, respectively (**Figure 1**).

### Survival Before and After PSM

**Figure 2:**
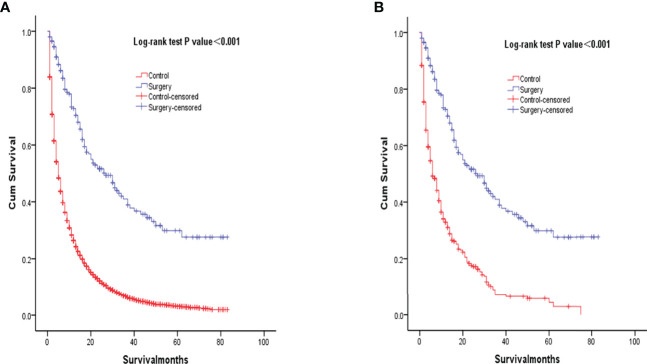
Kaplan-Meier overall survival curves of surgery-treated patients vs control before propensity score matching **(A)** and after propensity score matching **(B)**.

Kaplan‐Meier analysis showed that overall survival was significantly improved in patients who underwent surgery compared to the control group (P<0.001, **Figure 2A**). Median survival was 27 months for patients who underwent primary lung resection with mediastinal lymph node dissection at the start of the NSCLC diagnosis, compared with the control group. After matching patients undergoing surgical treatment with the propensity score, we balanced nearly all available covariates between groups, while a few covariates such as age, degree of tumor differentiation, tumor size, and tumor N-stage showed differences. After excluding the mismatched population, 203 surgical patients and 406 untreated patients were matched at 1:2 PSM (**Table 1**). To balance covariates, significant differences in survival time were also observed between patients treated surgically after PSM matching and those who did not receive treatment (P<0.001, **Figure 2B**).

**Table 1:** Summary characteristics of the overall sample stratified by surgery treatment before and after propensity score matching.

Variables	Before PSM			After PSM		
	Surgery (n = 203)	Control (n = 15500)	P value	Surgery (n = 203)	Control	P value	
					(n = 15500)		
Age (y)			<0.001			0.013	
≤65	140 (69.0)	8,623 (55.6)		140 (69.0)	238 (58.6)		
>65	63 (31.0)	6,877 (44.4)		63 (31.0)	168 (41.4)		
Sex			0.031			1	
Male (%)	89 (43.8)	7,979 (51.5)		89 (43.8)	178 (43.8)		
Female (%)	114 (56.2)	7,521 (48.5)		114 (56.2)	228 (56.2)		
Race			0.041			0.263	
White (%)	169 (83.3)	11,721 (75.6)		169 (83.3)	315 (77.6)		
Black (%)	18 (8.9)	2,063 (13.3)		18 (8.9)	49 (12.1)		
API (%)	16 (7.9)	1,716 (11.1)		16 (7.9)	42 (10.3)		
Marital status			0.620			0.774	
Married (%)	113 (55.7)	8,089 (52.2)		113 (55.7)	217 (53.4)		
Single and unmarried (%)	31 (15.3)	2,844 (18.3)		31 (15.3)	75 (18.5)		
Widowed, divorced, separated (%)	50 (24.6)	3,975 (25.6)		50 (24.6)	99 (84.4)		
Unclear (%)	9 (4.4)	592 (3.8)		9 (4.4)	15 (3.7)		
Insurance status			0.450			0.340	
Insured (%)	189 (93.1)	14,589 (94.1)		189 (93.1)	381 (93.8)		
Uninsured (%)	9 (4.4)	693 (4.5)		9 (4.4)	21 (5.2)		
Unclear (%)	5 (2.5)	218 (1.4)		5 (2.5)	4 (1.0)		
Histological type (%)			0.145			0.571	
Adenocarcinoma (%)	158 (77.8)	11,385 (73.5)		158 (77.8)	313 (77.1)		
Squamous cell carcinoma (%)	27 (13.3)	2,007 (12.9)		27 (13.3)	47 (11.6)		
NSCLC,adenosquamous carcinoma (%)	18 (8.9)	2,108 (13.6)		18 (8.9)	46 (11.3)		
Grade (differentiated)			<0.001			<0.001	
Well/Moderate (%)	73 (36.0)	2,001 (12.9)		73 (36.0)	63 (15.5)		
Poor/Undifferentiated (%)	113 (55.7)	4,672 (30.1)		113 (55.7)	121 (29.8)		
Unclear (%)	17 (8.4)	8,827 (56.9)		17 (8.4)	222 (54.7)		
AJCC N, 7th ed			0.014			0.069	
N0 (%)	97 (47.8)	2,800 (18.1)		97 (47.8)	68 (16.7)		
N1-N3 (%)	78 (38.4)	10,547 (68.0)		78 (38.4)	278 (68.5)		
Unclear (%)	28 (13.8)	2,153 (13.9)		28 (13.8%)	60 (14.8)		
Tumor size			<0.001			<0.001	
≤5cm (%)	125 (61.6)	6,539 (42.2)		125 (61.6)	180 (44.3)		
>5cm (%)	50 (24.6)	6,808 (43.9)		50 (24.6)	166 (40.9)		
Unclear (%)	28 (13.8)	2,153 (13.9)		28 (13.8)	60 (14.8)		
Chemotherapy			0.759			0.724	
Yes (%)	123 (60.6)	9,227 (59.5)		123 (60.6)	252 (62.1)		
No (%)	80 (39.4)	6,273 (40.5)		80 (39.4)	154 (37.9)		
Radiotherapy			0.612			0.953	
Yes (%)	163 (80.3)	12,240 (79.0)		163 (80.3)	322 (79.3)		
No (%)	36 (17.7)	2,766 (17.8)		36 (17.7)	75 (18.5)		
Unclear (%)	4 (2.0)	494 (3.2)		4 (2.0)	9 (2.2)		

### Prognostic Factors

**Table 2** lists the median survival results of univariate Kaplan-Meier analysis in the matched population. During surgery (P < 0.001), age (P < 0.001), sex (P = 0.010), marital status (P = 0.014), pathological type (P < 0.001), degree of differentiation (P= 0.001), tumor size (P < 0.001), lymph node (P < 0.001), chemotherapy (P < 0.001). There was significant difference in survival rate among covariables. Patients who did not undergo primary resection with mediastinal lymph node dissection, age >65 years, male, divorced, poorly differentiated or undifferentiated, tumor size >5cm, mediastinal lymph node metastasis, and did not undergo chemotherapy had poor survival. **Table 3** shows the multivariable predictors of mortality for the propensity matched sample. Patients in the propensity matching sample who underwent surgery had a significant reduction in mortality compared with those who did not (HR: 0.243, 95%CI: 0.162-0.365, P < 0.001). Age >65 years, squamous cell carcinoma, mediastinal lymph node metastasis, and no chemotherapy were independentlyassociated with higher mortality (P < 0.001, < 0.001 and < 0.001, **Table 3**). Mortality was higher in male, divorced, poorly differentiated or undifferentiated, and tumor size >5cm than in female, married, well-differentiated or moderate differentiated, and tumor size ≤5cm.

**Table 2:** Univariate analysis of prognostic factors for OS.

Variables	After PSM		
	All patients	Survival time (months), median (95% CI)	P value
Group			<0.001
Surgery	203	27 (19.332-34.668)	
Control	406	6 (4.709-7.291)	
Age (y)			<0.001
≤65	378	15 (12.688-17.312)	
>65	231	6 (4.368-7.632)	
Sex			0.010
Male	267	8 (5.821-10.179)	
Female	342	13 (10.600-15.405)	
Race			0.125
White	484	10 (8.297-11.703)	
Black	67	9 (6.941-11.059)	
Yellow	58	22 (17.064-26.936)	
Marital status			0.014
Married	330	14 (11.156-16.844)	
Single and unmarried	106	9 (6.161-11.839)	
Widowed, divorced, separated	149	8 (5.516-10.484)	
Unclear	24	13 (2.833-23.167)	
Insurance status			0.434
Insured	570	10 (8.118-11.882)	
Uninsured	30	12 (9.741-14.259)	
Unclear	9	20 (6.511-33.489)	
Histological type			<0.001
Adenocarcinoma	471	13 (10.846-15.154)	
Squamous cell carcinoma	74	5 (3.489-6.511)	
NSCLC, adenosquamous carcinoma	64	7 (2.712-11.288)	
Grade (differentiated)			0.001
Well/Moderate	136	16 (11.589-20.411)	
Poor/Undifferentiated	234	10 (7.238-12.762)	
Unclear	239	9 (7.313-10.687)	
Tumor size			<0.001
≤5cm	305	14 (11.612-16.388)	
>5cm	216	7 (5.371-8.629)	
Unclear	88	–	
TNM/N			<0.001
N0	165	16 (10.916-21.084)	
N1-N3	356	9 (7.168-10.832)	
Unclear	88	–	
Chemotherapy			<0.001
Yes	375	16 (13.872-18.128)	
No	234	4 (3.270-4.730)	
Radiotherapy			0.053
Yes	485	12 (10.112-13.888)	
No	111	6 (4.048-7.952)	
Unclear	13	4 (2.668-5.332)	

**Table 3:** Multivariate analysis of factors predictive of patients OS.

Variables	After PSM	
	HR (95% CI)	P value
Group, control (vs. surgery)	0.243 (0.162-0.365)	<0.001
Age, ≤65 (vs. >65)	1.620 (1.289-2.037)	<0.001
Sex, male (vs. female)	0.761 (0.620-0.934)	0.009
Race, white (vs. black)	1.026 (0.749-1.405)	0.873
Race, white (vs. API)	0.651 (0.453-0.936)	0.020
Marital status, Married (vs. Single and unmarried)	1.215 (0.916-1.611)	0.176
Marital status, Married (vs. Widowed, divorced, separated)	1.427 (1.116-1.825)	0.005
Insurance status, insured (vs. uninsured)	1.091 (0.694-1.713)	0.707
Histological type,Adenocarcinoma (vs. squamous cell carcinoma)	1.729 (1.290-2.318)	<0.001
Histological type,Adenocarcinoma (vs. NSCLC/Adenosquamous carcinoma)	0.960 (0.697-1.322)	0.804
Grade (differentiated),Well/Moderate (vs. poor/undifferentiated)	1.493 (1.141-1.954)	0.004
Tumor size, ≤5cm (vs>5cm)	1.263 (1.010-1.579)	0.040
TNM/N,N0 (vs. N1-N3)	1.716 (1.333-2.209)	<0.001
Chemotherapy, yes (vs. no)	3.041 (2.429-3.809)	<0.001
Radiotherapy, yes (vs. no)	0.913 (0.700-1.191)	0.503

### Predictors of Survival Among Surgery‐Treated Patients

Multivariate analysis assessed the predictors of survival in surgical patients, and the results were shown in **Table 4**. Patients >65 years of age who received surgical treatment had an increased risk of death compared with patients ≤65 years of age (HR= 1.587, 95%CI: 1.027-2.453, P= 0.034, **Table 4**). Patients who underwent surgery for squamous cell carcinoma had an increased mortality rate compared with those who underwent surgery for adenocarcinoma (HR= 2.009, 95%CI: 1.180-3.420, P = 0.010). Survival was significantly lower in patients undergoing chemotherapy than in patients not undergoing chemotherapy (HR = 2.555, 95%CI: 1.640-3.979, P < 0.001).

**Table 4:** Multivariate analysis of predictors of survival among patients with surgery patients.

Variables	HR (95% CI)	P value
Sex, male (vs. female)	0.66 (0.439-0.993)	0.149
Age, ≤65 (vs. >65)	1.587 (1.027-2.453)	0.034
Race, white (vs. black)	1.816 (0.942-3.499)	0.075
Race, white (vs. yellow)	0.676 (0.257-1.78)	0.428
Marital status, Married (vs. Single and unmarried)	1.584 (0.846-2.215)	0.188
Marital status, Married (vs. Widowed, divorced, separated)	1.369 (0.846-2.215)	0.125
Histological type,Adenocarcinoma (vs. squamous cell carcinoma)	2.009 (1.180-3.420)	0.010
Histological type,Adenocarcinoma (vs. NSCLC/Adenosquamous carcinoma)	0.998 (0.461-2.162)	0.996
Grade (differentiated),Well/Moderate (vs. poor/undifferentiated)	1.355 (0.882-2.080)	0.165
Tumor size, ≤5cm (vs>5cm)	1.034 (0.666-1.607)	0.881
TNM/T,T1-T2 (vs. T3-T4)	1.276 (0.794-2.052)	0.314
TNM/T,T1-T2 (vs. unclear)	1.198 (0.161-8.934)	0.86
TNM/N,N0 (vs. N1-N3)	1.388 (0.926-2.080)	0.113
TNM/N,N0 (vs. unclear)	0.496 (0.043-5.774)	0.575
Chemotherapy, yes (vs. no)	2.555 (1.640-3.979)	<0.001

### Subgroup Analysis After PSM

**Figure 3:**
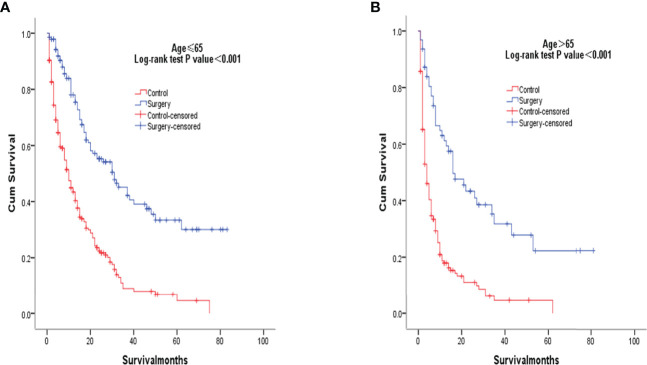
Kaplan-Meier overall survival curves of surgery-treated patients vs control after propensity score matching stratified by age ≤ 65years **(A)**, age> 65 years **(B)**.

**Figure 4:**
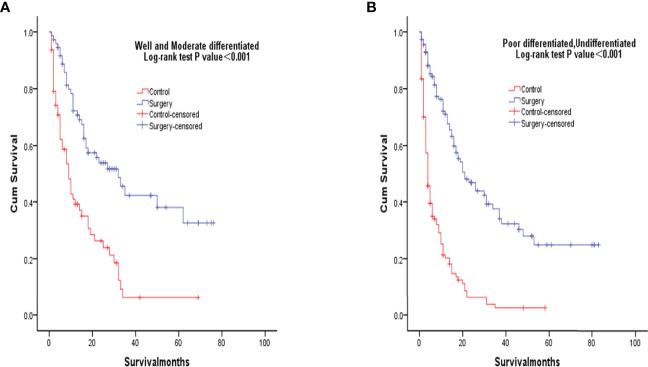
Kaplan-Meier overall survival curves of surgery-treated patients vs control after propensity score matching stratified by well and moderate **(A)**, poor and undifferentiated **(B)**.

**Figure 5:**
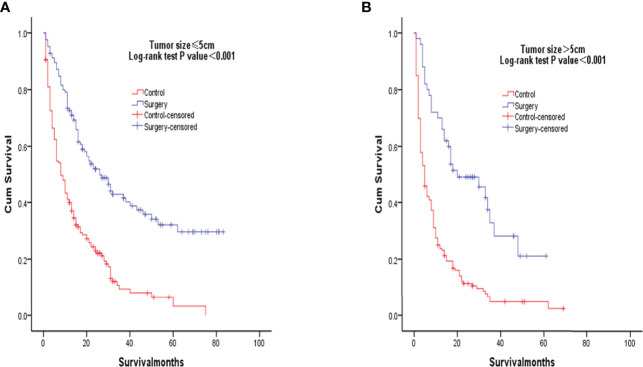
Kaplan-Meier overall survival curves of surgery-treated patients vs control after propensity score matching stratified by Tumor size, ≤5cm **(A)**, >5cm) **(B)**.

**Figure 6:**
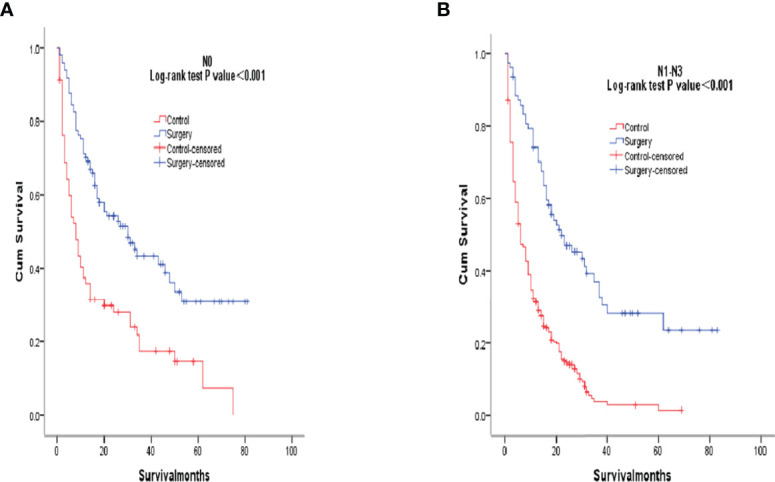
Kaplan-Meier overall survival curves of surgery-treated patients vs control after propensity score matching stratified by N0, **(A)**, N1-N3 **(B)**.

After PSM matching, differences in age, tumor differentiation, tumor size, and lymph nodes still existed between groups, so subgroup analysis was further performed. By age subgroup analysis, overall survival after PSM was longer in patients who underwent surgery than in patients who did not receive treatment (P < 0.001 and P < 0.001, **Figure 3**). In the subgroup of tumor differentiation degree, the survival rate of surgery group was significantly higher than that of untreated group (P < 0.001 and P < 0.001, **Figure 4**). In the tumor size subgroup, survival was significantly higher in the surgically treated group than in untreated patients (P < 0.001 and P < 0.001, **Figure 5**). Based on subgroup analysis of lymph nodes, patients who underwent surgery had significantly higher survival rates than untreated patients(P < 0.001 and P < 0.001, **Figure 6**).

## Discussion

In this study, radical pulmonary resection was independently associated with survival of NSCLC with BM. Primary lung tumor resection could improves survival in patients with BM from NSCLC.

Although relevant treatment guidelines for NSCLC with BM have been issued at home and abroad, there is still a lack of mature consensus due to the diversity and complexity of clinical symptoms and individual differences of patients, and there are still different views on the best treatment methods for concurrent BM of lung cancer in the academic circle. Simultaneous brain-lung resection is not widely used. In the early stage, it was believed that such patients did not benefit from the removal of the lung primary tumor, so they did not need active surgical treatment for the lung lesions.In recent years, for NSCLC patients with isolated single BM, selective resection of pulmonary lesions can improve the prognosis (13–18). Other studies have shown that SRS can also achieve better treatment effect for surgically resectable BM patients, and long-term survival is similar to surgery (7, 19–21). In addition, studies have suggested that whether complete resection of primary lung tumor is related to postoperative recurrence and prognosis (10, 11), but most cases are limited to selective local resection of lung lesions, and radical resection of lung lesions in NSCLC patients with BM is rarely reported on prognosis. HAN et al. (22) believed that lung surgery should still follow the principle of radical treatment and maximize the removal of tumor tissue and routine dissection of regional lymph nodes to achieve the best therapeutic effect. However, due to the small number of cases, the impact of radical surgical treatment on survival and prognosis of NSCLC patients combined with BM has not been fully discussed. In this study, all 203 patients underwent pulmonary primary tumor resection and mediastinal lymph node dissection, which confirmed that this radical surgical method of pulmonary primary tumor resection can prolong survival and have certain long-term survival benefits, and is an independent risk factor for OS.

Previous studies have shown that in addition to surgery, gender, race, adenocarcinoma, marital status, insurance status, mediastinal lymph nodes and other factors are also related to the prognosis of NSCLC patients with BM (19, 23–28). It has also been reported (22, 29) that the prognosis of lung surgery in NSCLC patients with BM is not related to mediastinal lymph node stage and T stage of lung lesions (11, 22). In this study, we performed PSM to minimize selection bias and make no significant differences in these factors when using the same sex, race, marital status, insurance status, histological type, and mediastinal lymph node subsets. So, the patients are evenly distributed. Our results suggest that radical resection of pulmonary lesions, youth (≤65 years), adenocarcinoma, and absence of mediastinal lymph node metastasis are independent prognostic factors for BM patients. Moreover, male, non-Asian race, divorce, poor tumor differentiation, and tumor >5cm were found to be negatively correlated predictors of prognosis in NSCLC patients with BM, which has been widely discussed in numerous studies (24, 29–32). In addition, BM suggests blood metastasis of tumor cells in patients, so it is necessary for NSCLC patients with BM to undergo chemotherapy (33, 34).The results of this group of cases showed that chemotherapy was an independent factor affecting prognosis. Although our study found no significant difference in survival risk between patients who received radiotherapy and those who did not, this may be due to the fact that the radiotherapy site (lung or brain) was not recorded for subgroup analysis. However, we believe that radiotherapy and chemotherapy play an indispensable role in the comprehensive treatment of NSCLC patients with BM.

### Limitations

There are some limitations in this study, which may affect our research results to some extent. The SEER database did not record data on patient complications, smoking history, and physical conditions. It is well known that some factors such as the number of BM, genetic changes and the treatment of BM affect the prognosis and clinical efficacy of patients (30, 35, 36), but the SEER database does not provide relevant information. These limitations may have some effect on overall prognosis, and we hope to refine this section in future prospective studies. Although the database contains specific radiotherapy and surgical methods, the lack of information on specific chemotherapy schemes has a certain impact on surgical evaluation, which is also the limitation of this paper. Therefore, we need more detailed data to assess the efficacy and adverse effects of surgery. Finally, although PSM was used to reduce selection bias in the surgical group, the retrospective nature of this study makes it difficult to avoid bias in other confounding factors.

## Conclusion

Based on our findings, it is evident that radical resection of primary lung can improve the survival of NSCLC patients with BM. In the future, a study with well-designed, multi-center, prospective randomized design is needed to validate this conclusion.

## Data Availability Statement

The datasets presented in this study can be found in online repositories. The names of the repository/repositories and accession number(s) can be found in the article/supplementary material.

## Ethics Statement

Ethical review and approval was not required for the study on human participants in accordance with the local legislation and institutional requirements. Written informed consent for participation was not required for this study in accordance with the national legislation and the institutional requirements.

## Author Contributions

QW performed the majority of experiments, acquisition of data, analysis and interpretation of data, statistical analysis, and drafting of the manuscript; JL and XL conducted part of the experiment, analysis and interpretation of data; QZ for critical revision of the manuscript for important intellectual content and study supervision.

## Funding

This work was supported by The National Natural Science Foundation of China (grant numbers 82103640 to JL), and The Natural Science Foundation of Shanghai (grant numbers 22ZR1409300 to JL).

## Conflict of Interest

The authors declare that the research was conducted in the absence of any commercial or financial relationships that can be interpreted to mean that there is no potential conflict of interest.

## Publisher’s Note

All claims expressed in this article are solely those of the authors and do not necessarily represent those of their affiliated organizations, or those of the publisher, the editors and the reviewers. Any product that may be evaluated in this article, or claim that may be made by its manufacturer, is not guaranteed or endorsed by the publisher.
